# Identifying risk factors for depression and positive/negative mood changes in college students using machine learning

**DOI:** 10.3389/fpubh.2025.1606947

**Published:** 2025-07-09

**Authors:** Qi Qiang, Jinsheng Hu, Xianke Chen, Weihua Guo, Qingshuo Yang, Zhijun Wang, Zhihong Liu, Ya Zhang, Qi Li

**Affiliations:** Department of Psychology, Liaoning Normal University, Dalian, China

**Keywords:** machine learning, college students, prediction, depression, change

## Abstract

**Background:**

In this study, machine learning was used to assess the prediction of the magnitude of depression changes in college students based on various psychological variable information.

**Methods:**

A group of college students from a certain school completed two assessments in October 2021 and March 2022, respectively. We collected baseline levels of depression, demographic variables, parenting styles, college students’ mental health information, personality information, coping styles, SCL-90, and social support information. We applied logistic regression, random forest, support vector machine, and k-nearest neighbor machine learning methods to predict the magnitude of depression changes in college students. We selected the best-performing model and outputted the importance of features collected at different time points.

**Results:**

Whether it is predicting the magnitude of positive changes or negative changes in depression, support vector machines (SVM) had the best prediction performance (with an accuracy of 89.4% for predicting negative changes in depression and an accuracy of 91.9% for predicting positive changes in depression). The baseline level of depression, father’s emotional expression, and mother’s emotional expression were all important predictors for predicting the negative and positive changes in depression among college students.

**Conclusion:**

Machine learning models can predict the extent of depression changes in college students. The baseline level of depression, as well as the emotional state of both fathers and mothers, play a significant role in predicting the negative and positive changes associated with depression in college students. This provides new insights and methods for future psychological health research and practice.

## 1 Introduction

Depression is a common mental illness that severely affects the quality of life and work ability of patients, and can even lead to serious consequences like suicide. With the development of social economy and the acceleration of pace of life, the incidence of depression has been increasing year by year, becoming an important issue in the field of global mental health. According to the “2022 National Depression Blue Book” data statistics, currently, 95 million people in China suffer from depression, and 50% of them are students. In terms of age distribution, patients under 18 years old account for 30.28%, those aged 18–24 account for 35.32%, those aged 25–30 account for 16.82%, those aged 31–40 account for 13%, those aged 41–50 account for 2.75%, and those over 50 years old account for 2.33%. From this data, it is evident that the age of depression patients in China is trending younger, with a relatively high proportion of students. Notably, the 18–24 age group, mainly composed of college students, is the largest and thus warrants special attention. Depression among college students has a negative impact on their individual well-being. For contemporary college students, regardless of the cause, depression significantly affects their learning and daily lives. It can induce melancholy, leading to mental and physical exhaustion, reduced interest in classes, and ultimately, academic failure in a vicious cycle.

The development and changes of depression are heterogeneous (1, 2), indicating that depressive symptom trends can vary among individuals. For some individuals, the depressive state may fluctuate over time, while for others, it may be stable and persistent. Because individuals with different patterns of depressive development may have different adverse outcomes, it is important to have specific treatment and care plans tailored to these variations. Thus, a better understanding of these changes can facilitate the effective allocation of medical resources. Particularly, individuals with severe depressive symptoms often have accompanying mental health issues (3, 4). Hence, it is necessary to establish a predictive model for the changes in depression among college students. Such a model can not only help identify those at risk of experiencing an exacerbation of depressive symptoms, allowing for early intervention and prevention of the progression of depression, but also reduce the severity of the condition and mitigate the negative impact on individuals’ lives and academic performance. Additionally, the model can assist in early identification of students whose depressive symptoms gradually decrease, as these students may still require ongoing psychological support and intervention to ensure long-term stability and recovery.

Previous research on depressive symptom development has mainly focused on diagnosed depression patients or normal individuals (5, 6). The classification of individual states was often limited to depressive and non - depressive, assuming a binary categorization (7). However, empirical studies have shown that individual depression is not a simple presence - or - absence matter, and depressed individuals may still exhibit qualitative differences (8). Additionally, previous studies predicting changes in depression have often focused on adolescents (9–11), the older adult (12–15), and postpartum women (16), with relatively fewer studies focusing on the dynamic changes in depression among college students. Furthermore, these types of studies often predict the trend of depression changes at a few specific time points, such as two (17) or more time points (18, 19). However, they often provide only a global prediction of the depressive state, lacking a thorough assessment of the specific magnitude and severity of depression changes. While these studies are crucial for early intervention and identifying potential risk factors for dynamic changes in depression, they still lack a comprehensive understanding of the range of depressive symptom changes. Therefore, shifting the focus from predicting depressive changes to predicting the magnitude of depressive changes can provide higher accuracy and more personalized guidance. Predicting the magnitude of depression changes can not only help determine the individual’s current depressive state and the changes in depressive symptoms but also capture the trends of varying degrees and directions of depression changes. This, in turn, can provide more accurate and targeted intervention recommendations.

There are many factors that influence the changes in depressive symptoms among college students, such as gender (20, 21), negative life events (22), family factors (23, 24), lifestyle (25), and environmental factors (26). The online psychological assessment system for college students in universities contains abundant individual-related, social relationship, and environmental information, which helps us to comprehensively understand the factors that affect depression among college students. However, there is currently no research on the impact of these factors on the magnitude of depression changes. By understanding the influence of different factors on the magnitude of depression changes among college students, we can gain a better understanding of the developmental patterns and mechanisms of depression among college students, which can contribute to improving the accuracy and effectiveness of diagnosis and intervention strategies for depression.

Furthermore, machine learning (ML) has been widely applied in research on predicting depression dynamic changes (27). ML can handle more variables and more complex variable associations than traditional regression methods (28), helping to discover specific depression - related feature patterns and quantify influencing factors. Therefore, we can utilize machine learning techniques to build predictive models for predicting the magnitude of depression changes.

Therefore, this study utilizes longitudinal assessment data from an online psychological assessment system for college students to evaluate the performance of different machine learning algorithms, including random forest (RF), k-nearest neighbors (k-NN), support vector machines (SVM), and logistic regression (LR). The aim is to build a predictive model for the magnitude of depression changes among college students and explore the best risk factors that influence the development of depression among them.

## 2 Methods

The data used in this study was obtained from an online psychological assessment system for college students at a certain university. This system provides a convenient and accessible way for students to assess their emotional well-being, identify potential mental health issues, and seek appropriate support. The study participants consisted of 5,534 undergraduate and graduate students from a certain university in the 2021 academic year. We took October 2021 as the baseline time point and conducted the first data collection. Six months later, we conducted the second data collection. We divided the participants into four groups based on their scores on the Beck Depression Inventory (BDI) at each time point. These groups are the healthy group (BDI < = 13), mild depression group (13 < BDI < = 19), moderate depression group (19 < BDI < = 28), and severe depression group (BDI > 28). Based on the division of the participants into these four groups at two time points, we further categorized the data into two overall groups: the negative change group (increase in depression severity) and the positive change group (decrease in depression severity).

We collected information from several questionnaires, including the Parental Bonding Instrument (PBI), Eysenck Personality Questionnaire (EPQ), Simplified Coping Style Questionnaire (SCSQ), Social Support Rating Scale (SSRS), University Personality Inventory (UPI), and Symptom Checklist-90 (SCL-90).

### 2.1 Research subjects and data collection

This study was approved by the school’s ethics review committee. A total of 5,534 full - time freshmen from a certain university were recruited through online channels. The specific process was as follows:

First, recruitment announcements were posted on platforms such as the school’s official website notice board, official WeChat public account, Weibo, and campus forum. The scope of promotion was expanded through class groups and club groups. The announcements detailed the research purpose, participation methods, privacy protection measures, and estimated time consumption. Students completed the registration by filling in information such as name (anonymous option available), student ID, school, major, and grade through the online registration system. The research team conducted a preliminary screening of the applicants and sent electronic informed consent forms to eligible students. After the participants confirmed their consent, they entered the formal research phase. Real - time technical support was provided during the research, and a secure platform was used to encrypt and store the data. After the study was completed, participants were offered small gifts or electronic certificates as a token of gratitude, and a brief summary of the research results was provided to them.

Among the 5,534 participants in this study, in terms of gender distribution, there are 1,392 males, accounting for 25.2%, and 4,142 females, accounting for 74.8%. Regarding the location of their family residence, 3,990 participants are from urban areas, accounting for 72.1%, and 1,544 are from rural areas, accounting for 27.9%.

The educational attainment of their parents is as follows: For the fathers, 41 have never attended primary school, accounting for 0.7%; 673 have a primary school education, accounting for 12.2%; 1,906 have a junior high school education, accounting for 34.4%; 1,533 have a senior high school or secondary vocational school education, accounting for 27.7%; 1,222 have a junior college or undergraduate education, accounting for 22.1%; and 159 have a postgraduate education, accounting for 2.9%. For the mothers, 82 have never attended primary school, accounting for 1.5%; 764 have a primary school education, accounting for 13.8%; 1,884 have a junior high school education, accounting for 34.0%; 1,561 have a senior high school or secondary vocational school education, accounting for 28.2%; 1,168 have a junior college or undergraduate education, accounting for 21.1%; and 75 have a postgraduate education, accounting for 1.4%.

### 2.2 Predictor variables

The predictor variables include: Sex, Age, Mother’s Care, Mother’s Autonomy, Mother’s Control, Father’s Care, Father’s Autonomy, Father’s Control, Objective Support, Subjective support, Extraversion, Neuroticism, Psychoticism, Lie scale, Positive Coping, Negative Coping, University Personality Inventory, Somatization, Obsessive-Compulsive, Interpersonal Sensitivity, Depression, Anxiety, Hostility, Phobic Anxiety, Paranoid Ideation, Psychoticism, Other.

### 2.3 Outcome variable

The outcome variables are the magnitude of negative change in depression and the magnitude of positive change in depression. Based on the level of negative change within the negative change group, we divided the magnitude of negative change in depression into three categories: one level of negative change (from healthy to mild depression, from mild depression to moderate depression, from moderate depression to severe depression), two levels of negative change (from healthy to moderate depression, from mild depression to severe depression), and three levels of negative change (from healthy to severe depression). The classification of the magnitude of positive change in depression in the positive change group follows the same logic as the negative change group, except for a decrease in the severity of depression.

Our classification is based on the theory of complex dynamic systems. In a dynamic system, state transitions at the global level may be sudden and discontinuous (29–31). The transition of depressive states is similar to the evolution of attractor states. The attractor measures the resilience of the system after being disturbed, and its state changes continuously from a stable state to an unstable state (29). When it is stable, the system has strong anti-interference and self-regulating abilities, while when it is unstable, it is prone to state transitions. Specifically, taking the classification of the magnitude of negative changes in depression into three levels as an example, a one-level negative change includes transitions such as “healthy → mildly depressed,” “mildly depressed → moderately depressed,” and “moderately depressed → severely depressed.” This corresponds to a small offset of the attractor, and the system remains relatively stable even under mild disturbances. For example, when transitioning from being healthy to mildly depressed, the individual has mild symptoms and can recover through self-regulation or simple interventions.

A two-level negative change includes transitions like “healthy → moderately depressed” and “mildly depressed → severely depressed.” This indicates a significant displacement of the attractor, and the stability of the system drops substantially. For instance, when transitioning from mild depression to severe depression, the individual has severe symptoms and requires professional intervention to restore stability. A three-level negative change is the transition of “healthy → severely depressed.” This reflects a drastic jump of the attractor, and the system is highly unstable. The individual has extremely severe symptoms and needs intensive treatment to reconstruct the psychological system. This classification system has clear guiding value for diagnosis and treatment. A one-level change serves as an early warning, indicating that closer observation and basic interventions are required. A two-level change provides a basis for adjusting the treatment plan. A three-level change guides major clinical decisions, covering emergency comprehensive treatment for severe depression and rehabilitation planning. In addition, by analyzing clinical data, this system can accurately predict the prognosis of patients, significantly enhancing the pertinence and effectiveness of clinical interventions.

### 2.4 Data preparation

#### 2.4.1 Data cleaning

During the data collection process, a forced - response online collection system was adopted, which effectively avoided the traditional problem of missing values. For duplicate values, by comparing the combinations of user IDs, timestamps, and key features, the drop_duplicates() function in the pandas library of Python was used to identify and remove duplicate records, ensuring the uniqueness of the data. In terms of outlier handling, box plots were used for detection, with the inter - quartile range (IQR) as the judgment basis. If the data exceeded Q1–1.5 × IQR or Q3 + 1.5 × IQR, they were regarded as mild outliers and adjusted to the corresponding quantile thresholds. If the data exceeded Q1–3 × IQR or Q3 + 3 × IQR, they were identified as extreme outliers and replaced with the maximum or minimum boundary values. This ensured that the distribution characteristics of the data were retained while improving the robustness of the model.

#### 2.4.2 Data balancing

To address the issue of data imbalance, the SMOTE (Synthetic Minority Over-sampling Technique) method (32) was employed. By synthesizing samples of the minority class, the number of samples in each class was made relatively balanced, thus enhancing the model’s learning effect for the minority class samples.

#### 2.4.3 Feature transformation

In terms of feature encoding, for categorical variables such as gender, the One-Hot Encoding technique was applied. With the help of the OneHotEncoder in the scikit-learn library, vectorization transformation was achieved, converting categorical information into a numerical form that can be processed by the model. For continuous variables such as age and scale scores, the *Z*-score standardization method (\(z = \frac{x - \mu}{\sigma}\)) was adopted. The StandardScaler was used to normalize the data, making its mean value 0 and standard deviation 1, thus eliminating the dimensional differences between different features.

#### 2.4.4 Feature selection

The Randomized Lasso Algorithm was used. This algorithm is an improvement of the traditional Lasso algorithm and can effectively solve the stability problem of the traditional Lasso algorithm in feature selection (33). Through this algorithm, a feature subset that makes a greater contribution to the model was screened out to optimize the model’s performance. After feature selection, in the model for predicting the magnitude of positive changes in the depression level, four features were filtered out. However, in the model for predicting the magnitude of negative changes in the depression level, all features were retained.

### 2.5 Statistical analysis

#### 2.5.1 Data description

We conducted descriptive statistical analysis for all the predictor variables. Chi-square tests and one-way analysis of variance (ANOVA) were used to analyze the differences in predictor variables among different change level groups based on the type of variables. All tests were two-tailed, and a significance level of *p* = 0.05 was set. Statistical analysis was performed using SPSS 26.0.

#### 2.5.2 Model tuning and model comparison

We utilized four machine - learning algorithms: logistic regression, support vector machine, random forest, and K - nearest neighbors. To optimize the performance of these algorithms, we employed grid search in a 10 - fold cross - validation framework. The random state was set to 42 (random_state = 42) to ensure reproducibility. The detailed information about the hyperparameter grid search can be found in Table 1. After determining the optimal parameter combinations based on the average accuracy scores from the cross - validation, we once again applied a 10 - fold cross - validation method to evaluate the performance of the predictive models with the selected parameters (34). The performance was measured using several metrics, including accuracy, F1 score, precision, area under the curve (AUC), specificity, and sensitivity. The performance metrics include overall accuracy, balanced accuracy, F1 score, precision, area under the curve (AUC), specificity, sensitivity and Cohen’s kappa.

**Table 1:** The details of the hyperparameter grid search.

Machine learning algorithms	Parameters and their ranges
LR	C[10^−3^, 10^−2^, 10^−1^, 1, 10, 10^2^, 10^3^]; penalty [‘l1’, ‘l2’]; solver [‘newton-cg’, ‘lbfgs’, ‘liblinear’, ‘sag’, ‘saga’]
SVM	C[10^−3^, 10^−2^, 10^−1^, 1, 10, 10^2^, 10^3^]; gammaC [10^−3^, 10^−2^, 10^−1^, 1, 10, 10^2^, 10^3^]
RF	n_estimators[50, 100, 150, 200]; max_depth[“none,"10, 50, 100]; min_samples_split[10, 20, 30]; min_samples_leaf[10, 20, 30]
k-NN	k[1, 3, 5, 7, 9];weights[“uniform,” “distance”];metric[“euclidean,” “manhattan”]

SVM, Support Vector Machine; LR, Logistic Regression; RF, Random Forest; k-NN, k-Nearest Neighbors.

#### 2.5.3 Determinant importance

Based on the results of model comparison, we generated the output of important features for the optimal model. In the machine learning analysis, all data analysis was conducted using Python 3.7 and scikit-learn v22.2 (35).

**Figure 1:**
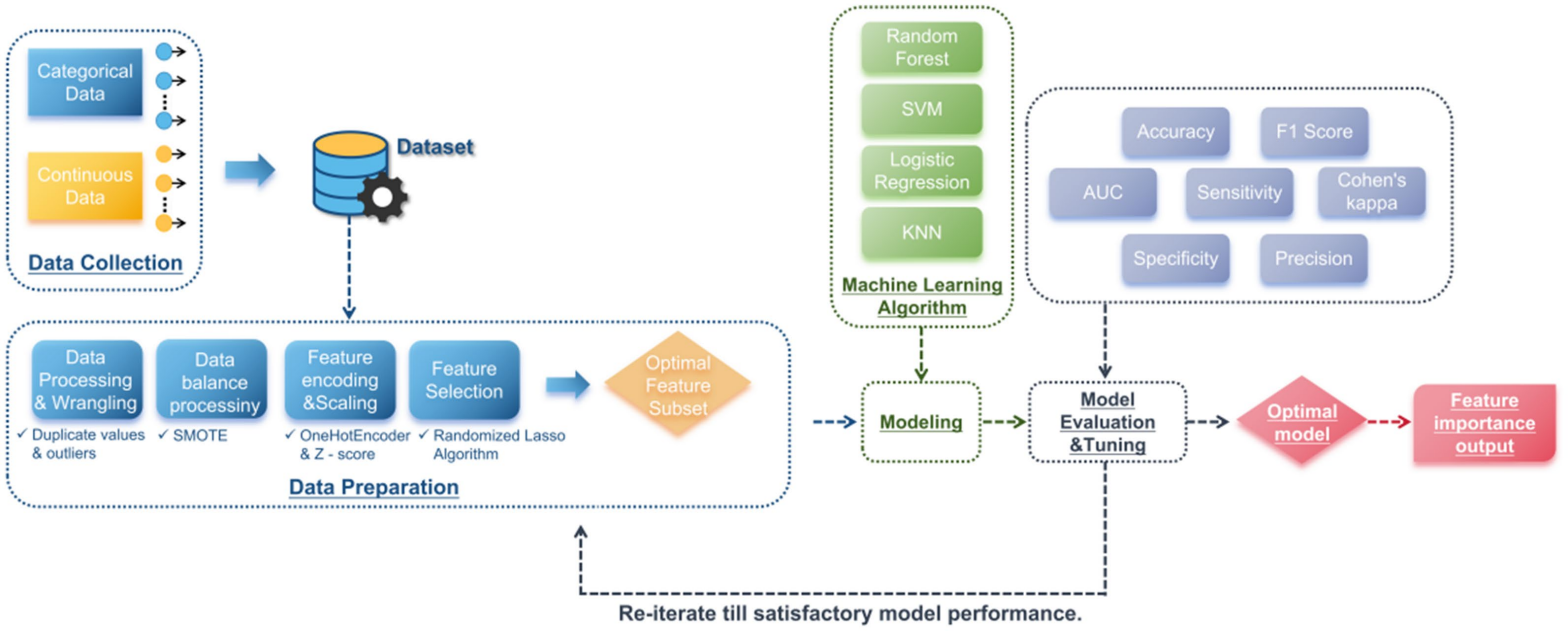
Data processing and modeling workflow diagram.

Figure 1 shows the overall process of data from collection to model construction and evaluation.

## 3 Results

The results of descriptive statistics (Supplementary Table 1) indicate that there were a total of 370 cases of negative changes in depression levels among college students. Among them, 62.16% showed one level of negative change (including changes from healthy to mild, from mild to moderate, and from moderate to severe), 28.10% showed two levels of negative change (including changes from healthy to moderate and from mild to severe), and 9.74% showed three levels of negative change (including change from healthy to severe).

The results of one-way ANOVA and chi-square tests showed significant differences in the negative changes of depression levels among college students in terms of variables Sex, Baseline Depression, Mother’s Care, and Mother’s Autonomy.

Supplementary Table 2 shows the descriptive statistics of positive changes in depression levels among college students. The total number of positive changes in depression level was 547, of which 61.97% showed one level of positive change (including changes from mild to healthy, from moderate to mild, and from severe to moderate), 30.71% showed two levels of positive change (including changes from moderate to healthy and from severe to mild), and 7.32% showed three levels of negative change (including change from severe to healthy).

The results of one-way ANOVA and chi-square tests showed significant differences on various dimensions of Baseline Depression, Mother’s Care, Neuroticism-Psychoticism, Positive Coping, University Personality Inventory, and SCL-90 scale in positive changes of depression levels among college students.

This study aims to utilize four machine learning algorithms to predict the magnitude of negative change in depression levels among college students (Table 2). The Support Vector Machine (SVM) clearly outperformed the others. It boasted an overall accuracy of 0.894, an F1 - score of 0.892, and a precision of 0.895. With an AUC of 0.967, it showed excellent classification ability. Its high specificity (0.946) and sensitivity (0.896) along with a balanced accuracy of 0.895 and Cohen’s kappa of 0.839 indicate consistent and reliable performance. The k - Nearest Neighbors (k - NN) algorithm ranked second, achieving an overall accuracy of 0.789. While its precision of 0.810 was relatively high, its specificity was 0.894. The balanced accuracy was 0.789 and Cohen’s kappa was 0.682. Random Forest (RF) had an overall accuracy of 0.723. Its AUC of 0.895 was decent, but other metrics were less impressive compared to the top two. Logistic Regression (LR) performed the worst, with an overall accuracy of just 0.579. Its low Cohen’s kappa of 0.371 suggested limited agreement between predictions and actual results. Overall, SVM is the most effective for this prediction task.

**Table 2:** Comparative results of multiple models for predicting the magnitude of negative change in depression among college students.

Model	Overall Accuracy	F1-Score	Precision	AUC	Specificity	Sensitivity	Balanced accuracy	Cohen’s kappa
LR	0.579	0.570	0.577	0.775	0.792	0.585	0.585	0.371
SVM	0.894	0.892	0.895	0.967	0.946	0.896	0.895	0.839
RF	0.723	0.713	0.719	0.895	0.862	0.729	0.728	0.583
k-NN	0.789	0.768	0.810	0.842	0.894	0.790	0.789	0.682

AUC, area under the curve; LR, logistic regression; SVM, support vector machine; RF, random forest; k-NN, k-nearest neighbors.

Here are the results of four machine learning algorithms in predicting the magnitude of positive change in depression levels among college students (Table 3). The Support Vector Machine (SVM) demonstrated outstanding performance, achieving an overall accuracy of 0.919 and a balanced accuracy of 0.920, which highlighted its consistent performance across different classes. With an F1-score of 0.918, precision of 0.917, and an AUC of 0.983, it showed superior classification ability. Its Cohen’s kappa coefficient of 0.878 further confirmed the high agreement between predicted and actual results. Random Forest (RF) ranked second, attaining an overall accuracy of 0.906. Although its performance was commendable, metrics like precision (0.913) and AUC (0.972) were slightly lower than SVM’s, indicating a marginally less effective classification. Logistic Regression (LR) achieved an overall accuracy of 0.847. While it had a relatively high AUC of 0.940, its overall accuracy and other metrics were lower than the top two algorithms. The k-Nearest Neighbors (k-NN) algorithm performed the worst, with an overall accuracy of 0.816, suggesting significant limitations in predicting depression level changes.

**Table 3:** Comparative results of multiple models for predicting the magnitude of positive change in depression among college students.

Model	Overall Accuracy	F1-Score	Precision	AUC	Specificity	Sensitivity	Balanced accuracy	Cohen’s kappa
LR	0.847	0.843	0.844	0.940	0.924	0.846	0.846	0.770
SVM	0.919	0.918	0.917	0.983	0.960	0.920	0.920	0.878
RF	0.906	0.904	0.913	0.972	0.953	0.904	0.904	0.858
k-NN	0.816	0.802	0.833	0.861	0.908	0.815	0.815	0.723

AUC, area under the curve; LR, logistic regression; SVM, support vector machine; RF, random forest; k-NN, k-nearest neighbors.

**Figure 2:**
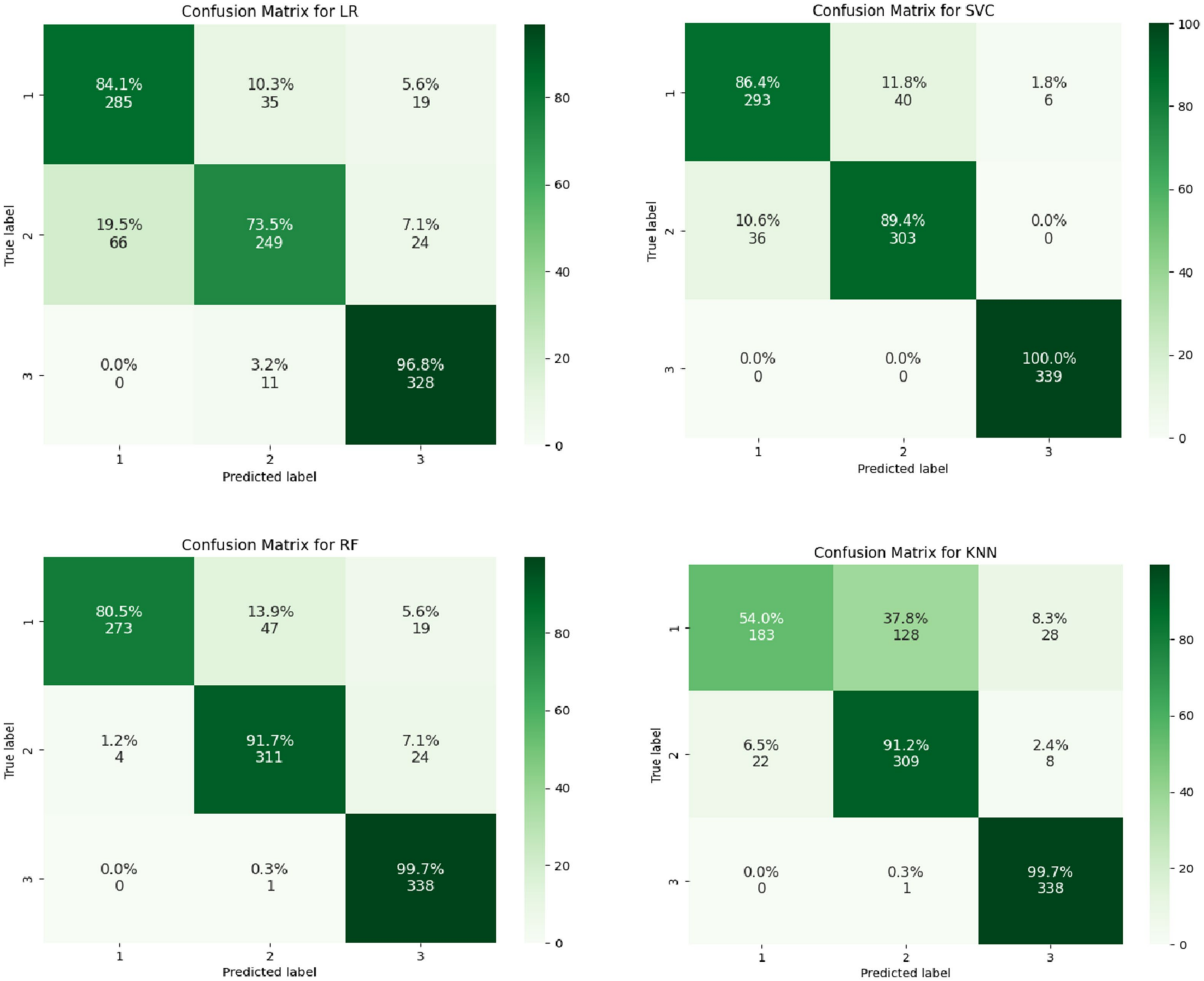
Confusion matrices of multiple models for predicting the magnitude of positive changes in college students’ depression.

**Figure 3:**
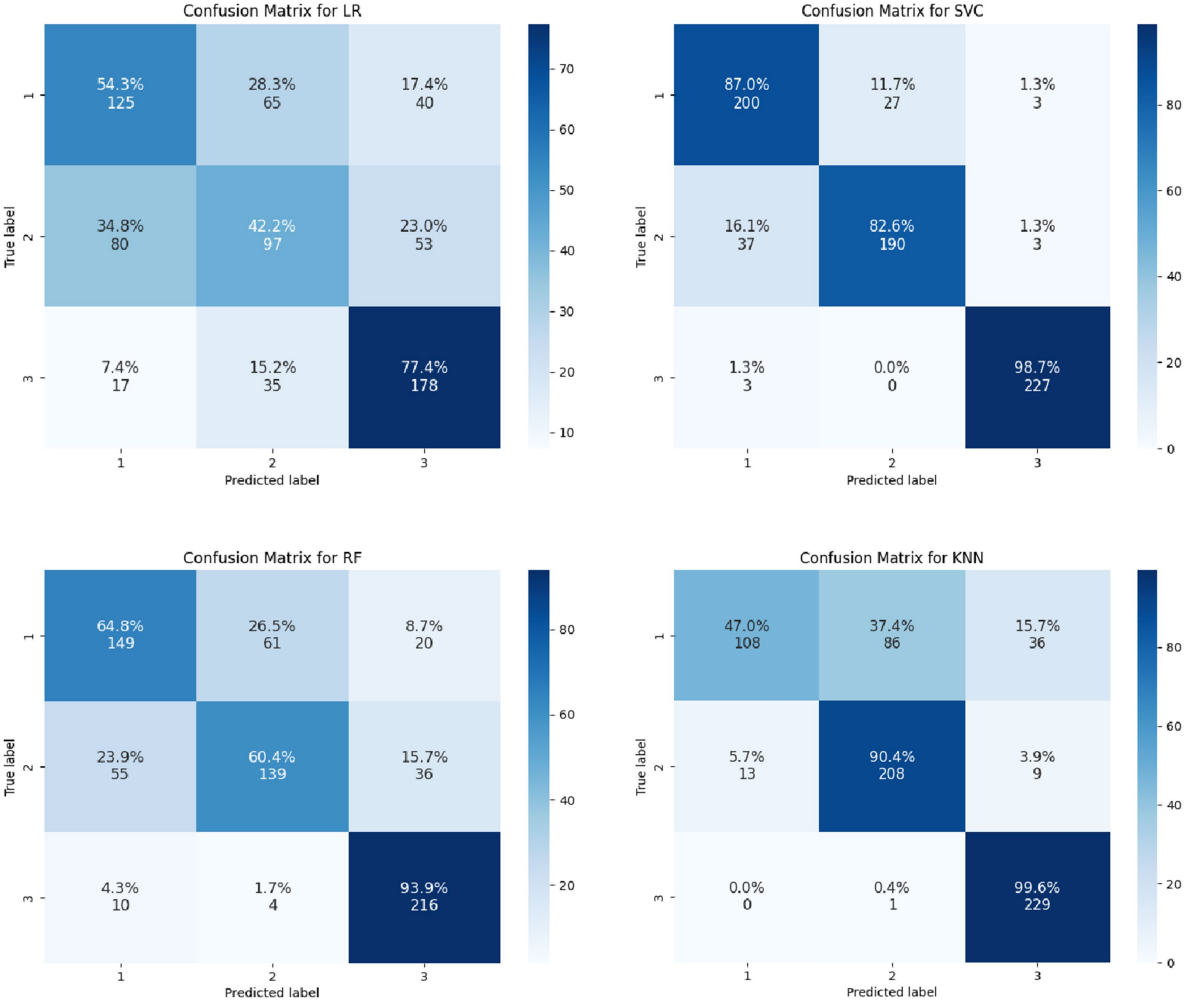
Confusion matrices of multiple models for predicting the magnitude of negative changes in college students’ depression.

From the output results of the confusion matrices (Figures 2, 3), whether it is for the identification of the categories of the magnitude of positive changes in the depression or the categories of the magnitude of negative changes in the depression, the SVC model overall performs the best in terms of the proportion of correct classifications. Overall, SVM proved to be the most effective algorithm for this prediction task.

**Figure 4:**
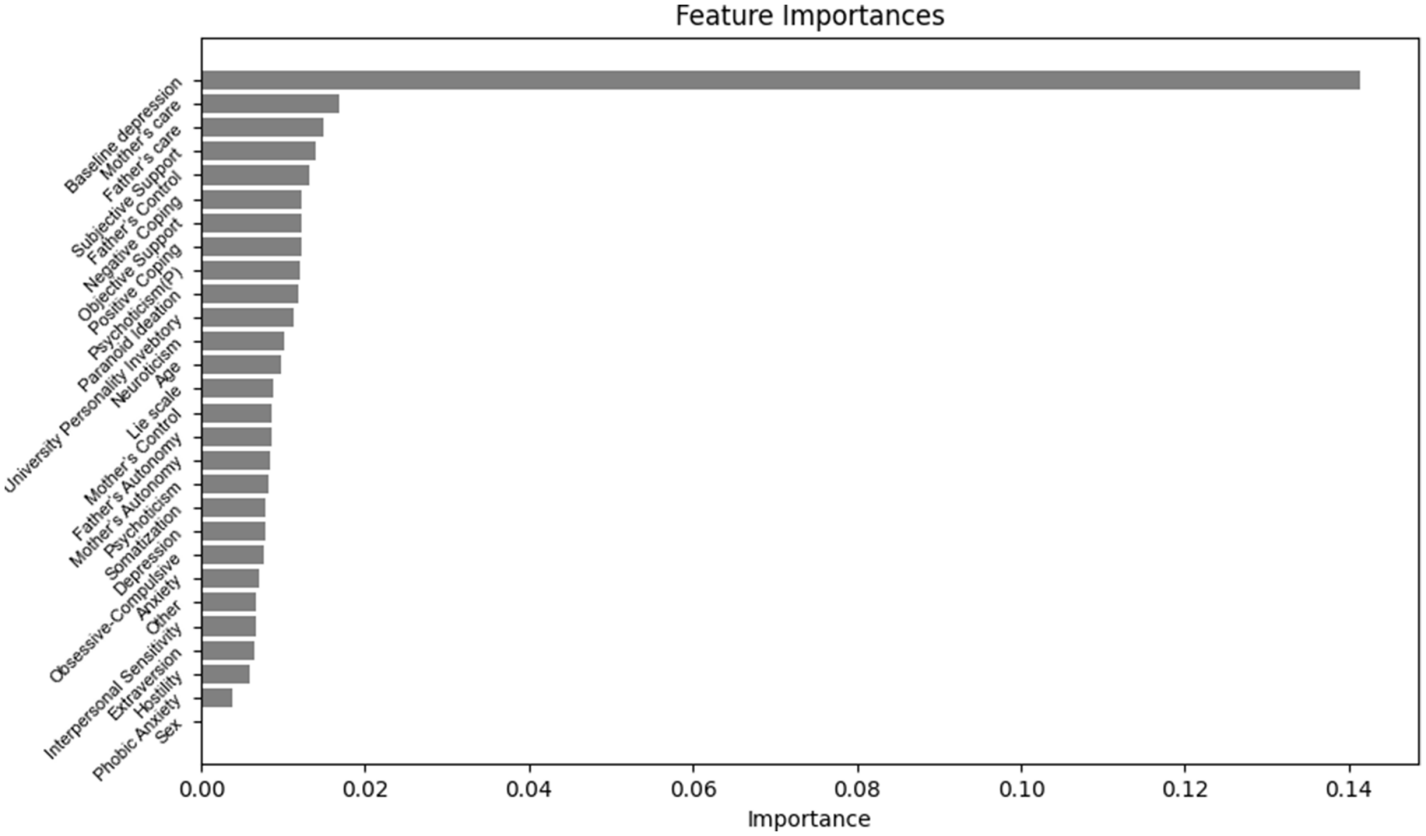
Importance ranking of features for predicting the magnitude of depressive negative change in university students.

The importance ranking of features in predicting the magnitude of negative change in depression levels among college students is as follows (Figure 4): baseline depression, Lie scale, University Personality Inventory, Father’s Care, Mother’s Care, Age, Objective Support, Positive Coping, Father’s Autonomy, Extraversion, Phobic Anxiety, Somatization, Sex, Hostility, Other, Obsessive-Compulsive, Subjective support, Mother’s Autonomy, Psychoticism, Father’s Control, Negative Coping, Anxiety, Neuroticism, Mother’s Control, Psychoticism, Depression, Paranoid Ideation, Interpersonal Sensitivity.

**Figure 5:**
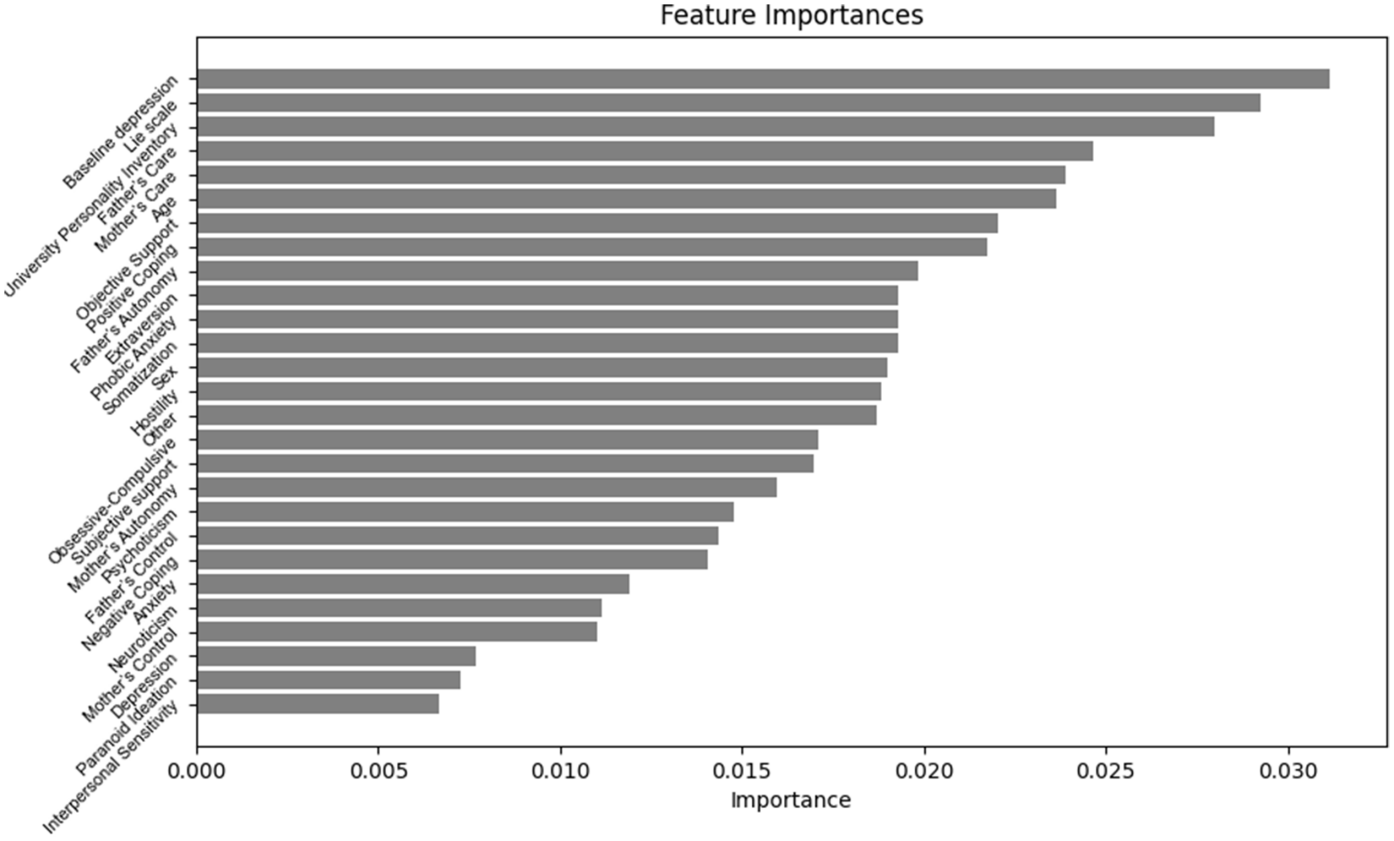
Importance ranking of features for predicting the magnitude of depressive positive change in university students.

According to our research, the importance ranking of features in predicting the magnitude of positive change in depression levels among college students is as follows (Figure 5): Baseline depression, Mother’s Care, Subjective support, Father’s Care, Father’s Control, Psychoticism, Negative Coping, Positive Coping, Objective Support, Paranoid Ideation, University Personality Inventory, Other, Somatization, Age, Extraversion, Psychoticism, Mother’s Autonomy, Father’s Autonomy, Depression, Mother’s Control, Lie scale, Hostility, Anxiety, Gender.

## 4 Discussion

In terms of gender distribution, the proportion of female students is relatively high, which may be related to the university’s disciplinary setup and enrollment situation. Regarding the location of family residence, the number of students from urban areas is slightly higher than that from rural areas. The educational attainment of parents shows a certain degree of diversity, but overall, those with a junior high school education or above are in the majority. Although this sample includes students with different genders, family residence locations, and parental educational backgrounds, since it is only from one university, there are limitations in terms of geographical diversity and the diversity of institution types. Such limitations may lead to certain biases when generalizing the research results to students from other institutions or different regions. However, the findings of this study can still provide valuable references for understanding the relevant characteristics of the student body of this university. Subsequent studies can consider expanding the sample scope and including students from more different types of institutions and regions to further enhance the representativeness and generalizability of the research results.

According to descriptive statistical results, the ratio between negative and positive changes in the magnitude of depression levels shows astonishing consistency. We found that for one level of negative or positive change, it is around 60%, while for two levels it is around 30%, and for three levels it is around 10%. These results indicate the complexity and diversity of depression, highlighting individual differences in the manifestation and variability of depressive symptoms. Moreover, the findings emphasize the uncertainty and variability in the changes of depression, implying that depression is not a static state but a dynamic process (36–37). The ratio of negative and positive changes in the magnitude of depression levels shows a consistent trend, demonstrating a relatively balanced distribution. This finding is important for gaining a deeper understanding of the process of depression, individual differences, and the development of personalized intervention measures.

Compared with previous studies that focused on the static prediction of depression or simple classification of it, this study innovatively divides the magnitude of depressive changes into three levels, namely the first, second, and third levels of changes (covering both positive and negative directions), and constructs a prediction model based on this classification. According to our results, our machine learning models supported the hypothesis that the magnitude of depression change can be classified. Both the classification of negative changes in depression magnitude and positive changes in depression magnitude appear to be reasonable, with SVM achieving an accuracy of over 85% in Tables 2, 3. Among them, support vector machines, random forests, and k-nearest neighbors machine learning models have higher accuracy in predicting the magnitude of negative changes in depression compared to the logistic regression model. (ACC: 89.4, 72.3, 78.9, and 57.9%). In contrast, support vector machines and random forests, two types of classifiers, have higher accuracy in predicting the magnitude of positive changes in depression compared to k-nearest neighbors and logistic regression.(ACC: 91.9, 90.6, 81.6, and 84.7%). Therefore, we have enriched the research on predicting depression changes from a more nuanced perspective, which helps us gain a deeper understanding of the developmental process and mechanisms of depression. By considering the magnitude of depression change, we can uncover hidden relationships and patterns, further broadening our knowledge of depression. This will provide more targeted guidance for the development of more effective intervention strategies and treatment methods.

The predictive factors identified by our machine learning models are essential for understanding the essence of depression magnitude in college students, whether predicting negative or positive changes. We found that the baseline level of depression, father’s care, and mother’s care are important predictive factors in predicting the magnitude of both negative and positive changes in depression among college students. Previous research has also found that baseline depression severity is only an important predictor for the negative change group (38). Our study enriches previous research by revealing that baseline depression level is also an important factor for positive changes in depression. This suggests that baseline depression level not only correlates with negative changes but also predicts the extent to which individuals experience positive changes. This provides deeper guidance and theoretical support for depression prevention and intervention. In our future research, we will further explore the reasons and mechanisms behind these differences and determine the ways and extent to which baseline depression level influences both negative and positive changes in depression. Additionally, parental care were also found to be important in influencing the magnitude of depression change in college students. Previous research has shown that Family health, family support, and family function play a crucial role in the psychological development of individuals (39–41). Integrating with prior research, we posit that parental care and support can mitigate college students’ depressive tendencies and enhance their emotional coping abilities, reducing the magnitude of negative depression changes. Furthermore, a higher level of maternal care may promote positive changes in college students, while more paternal care may facilitate positive changes as well.

However, the research findings may vary across different educational and cultural backgrounds. In China, traditional family concepts and the social environment have made family upbringing have a profound impact on children (42, 43). Parents’ extensive involvement in education has rendered it a crucial factor influencing the changes in children’s depression status. Influenced by individualistic culture, unique family structures, and educational concepts in the West (44, 45), children are more likely to rely on their own self-regulation to cope with emotional problems, and the influence of family factors may be correspondingly weakened. Therefore, it is urgent to explore cultural differences by using Western samples in the future. Multi-site verification can be carried out: establish an international cooperation network to collect data uniformly, combine qualitative methods such as in-depth interviews to uncover unique factors, conduct longitudinal tracking in multiple regions, and dynamically monitor the long-term relationship between predictive factors and changes in depression, so as to enhance the reliability and universality of the research conclusions.

The strength of this study lies in its pioneering use of machine learning methods to predict the magnitude of depression changes in college students and providing some important predictive factors. This helps deepen our understanding of the developmental process and mechanisms of depression, providing guidance for personalized interventions and treatments. Additionally, the research results highlight the potential application of machine learning in mental health, bringing new possibilities for personalized mental health management. However, the actual deployment of the model faces significant ethical challenges. The sensitivity of psychological data makes it prone to the risk of privacy leakage. The potential biases in the algorithm may lead to unfair group evaluations (46). False positive results will result in excessive intervention, while false negative results may delay the appropriate time for treatment. Regarding the possible issues of false negatives and false positives in the prediction results, we particularly emphasize that in practical application scenarios such as campuses, counselors and relevant practitioners need to maintain a cautious attitude. They should combine the model prediction results with traditional assessment methods such as qualitative research and interviews, and reduce the risk of misjudgment through comprehensive judgment. At the same time, we propose strategies such as multi-disciplinary review and the use of interpretable algorithms to ensure that the research is both scientific and humanistic, and to guarantee the reasonable application of the prediction model in real scenarios.

### 4.1 Limitations

This study also has some limitations that may introduce biases into the results.

Sample Limitations: The sample is only sourced from a single university. Although multiple recruitment channels were adopted to cover different groups, there are still deficiencies in regional representativeness and sample diversity, which may affect the generalization of the research conclusions to college students in other universities or regions. In addition, the online voluntary participation mode may lead to selection bias, and the characteristics of students who actively participate may differ from those who do not participate. Variable and Model Limitations: The predictive factors considered in the study and the machine learning models used may not be comprehensive enough. Although important predictive factors such as baseline depression level and parental care were included in the study, other factors such as the history of childhood trauma, peer relationships, physical activities, etc., may also have a significant impact on the changes in college students’ depression (47). Due to the lack of consideration of these factors, the research results may not be able to fully reveal the complex mechanisms of depression changes. Validation Limitations: Currently, the research conclusions are only based on a single dataset collected at two time points from the same institution, and the model performance is evaluated through internal cross-validation. Although internal cross-validation can verify the robustness of the model to a certain extent, the lack of external validation leads to insufficient universality of the research conclusions. External validation is a crucial step to test whether the research results can be replicated in different samples and environments. The absence of this step may limit the research conclusions to specific populations in specific institutions, making it difficult to generalize them to broader scenarios.

In response to the above limitations, future research can be carried out in the following directions: Optimize Sample Selection: Strengthen the representativeness and diversity of the sample by jointly recruiting from multiple universities, expanding the sample coverage, or using methods such as stratified sampling, and further verify the universality of the research conclusions. Improve Variables and Models: Incorporate more potential influencing factors, such as the history of childhood trauma, peer relationships, etc., improve the predictive model, and more comprehensively reveal the complex mechanisms of changes in college students’ depression. Strengthen External Validation: Cooperate with multiple universities and educational institutions in China, collect samples of college students covering different regions and cultural backgrounds, construct a joint dataset of multiple institutions, verify the model and method of this study, and evaluate the applicability of the research conclusions in diverse populations. In addition, the research objects can be expanded to different groups at different life stages, such as middle school students and new employees in the workplace, to explore the common laws of psychological characteristics and behavioral patterns, and verify the effectiveness of the theoretical model in different scenarios. Through multi-dimensional external validation and repeated research, it will help to improve the theoretical framework and enhance the universal value and practical guiding significance of the research results.

## 5 Conclusion

In summary, the results confirm the complexity, diversity, and dynamics of depression revealed by machine learning methods and descriptive statistical analysis, as well as the effectiveness of machine learning methods in predicting the magnitude of depression changes in college students. It emphasizes the importance of predictive factors in the process of depression magnitude changes. This provides new insights and methods for deepening our understanding of the developmental process and mechanisms of depression, developing personalized intervention measures, and future mental health research and practices.

## Data Availability

The original contributions presented in the study are included in the article/Supplementary material, further inquiries can be directed to the corresponding author.
