# Supplementary material for: Identifying risk factors for depression and positive/negative mood changes in college students using machine learning
Source: Front Public Health. 2025 Jul 9;13:1606947. doi: 10.3389/fpubh.2025.1606947 (PMC12283326; doi:10.3389/fpubh.2025.1606947)
Supplement: Supplementary file 1 [file Table_1.DOCX]

**Table 1.** Descriptive statistical results of the magnitude of negative change in depression among college students

| Variables | A negative change of one level（230,62.16%） | A negative change of two levels（104,28.10%） | A negative change of three levels（36,9.74%） | F\χ² | *p* |
| --- | --- | --- | --- | --- | --- |
|  | Mean (S.D.)\  *N* (%) | Mean (S.D.)\  *N* (%) | Mean (S.D.)\  *N* (%) |  |  |
| Sex |  |  |  |  |  |
| Male | 66(28.69%) | 39(37.50%) | 18(50.00%) | 7.54 | 0.023 |
| Female | 164(71.31%) | 65(62.50%) | 18(50.00%) |  |  |
| Age | 20.00（2.68） | 19.51（2.41） | 19.52（2.10） | 1.637 | 0.196 |
| Baseline Depression | 10.76（6.92） | 8.95（5.25） | 6.50（4.53） | 8.562 | <0.001 |
| Mother’s Care | 22.44(6.51) | 22.510(6.21) | 19.02(8.72) | 4.300 | 0.014 |
| Mother’s Autonomy | 10.49(4.09) | 10.77(4.06) | 8.55(4.99) | 3.975 | 0.020 |
| Mother’s Control | 6.14(2.81) | 6.24(2.70) | 6.13(3.15) | 0.045 | 0.956 |
| Father’s Care | 29.50(5.32) | 19.50(5.94) | 17.22(6.68) | 2.659 | 0.071 |
| Father’s Autonomy | 10.07(3.16) | 9.72(3.70) | 9.11(3.96) | 1.401 | 0.248 |
| Father’s Control | 5.95(2.65) | 5.77(2.70) | 5.55(2.61) | 0.419 | 0.658 |
| Objective Support | 11.07(3.68) | 11.41(3.83) | 11.36(4.21) | 0.326 | 0.722 |
| Subjective support | 19.80(4.12) | 20.27(4.31) | 20.00(4.78) | 0.441 | 0.644 |
| E（Extraversion） | 51.56(11.12) | 52.25(11.84) | 51.66(12.18) | 0.134 | 0.875 |
| N（Neuroticism） | 55.47(11.57) | 56.10(11.38) | 58.47(11.07) | 1.073 | 0.343 |
| P（Psychoticism） | 50.95(10.08) | 49.80(9.47) | 51.25(8.48) | 0.567 | 0.568 |
| L（Lie scale） | 43.73(9.03) | 44.08(8.72) | 45.27(9.92) | 0.460 | 0.632 |
| Positive Coping | 21.62(6.18) | 22.44(5.48) | 21.58(6.66) | 0.692 | 0.501 |
| Negative Coping | 10.38(4.09) | 11.19(4.51) | 10.58(4.61) | 1.292 | 0.276 |
| University Personality Inventory | 22.29(10.17) | 21.97(10.92) | 19.13(11.25) | 1.408 | 0.246 |
| Somatization | 1.59(0.60) | 1.64(0.63) | 1.54(0.55) | 0.385 | 0.680 |
| Obsessive-Compulsive | 2.39(0.75) | 2.41(0.76) | 2.15(0.68) | 1.766 | 0.173 |
| Interpersonal Sensitivity | 2.14(0.78) | 2.28(0.76) | 2.09(0.73) | 1.422 | 0.242 |
| Depression | 1.96(0.67) | 1.96(0.70) | 1.80(0.59) | 0.889 | 0.412 |
| Anxiety | 1.85(0.65) | 1.89(0.74) | 1.73(0.54) | 0.815 | 0.443 |
| Hostility | 1.77(0.71) | 1.86(0.75) | 1.54(0.61) | 2.688 | 0.069 |
| Phobic Anxiety | 1.71(0.69) | 1.71(0.70) | 1.60(0.63) | 0.650 | 0.523 |
| Paranoid Ideation | 1.84(0.71) | 1.93(0.70) | 1.83(0.60) | 0.628 | 0.534 |
| Psychoticism | 1.80(0.67) | 1.82(0.59) | 1.69(0.61) | 0.549 | 0.578 |
| Other | 1.71(0.61) | 1.68(0.53) | 1.63(0.60) | 0.372 | 0.689 |
