# Supplementary material for: Identifying risk factors for depression and positive/negative mood changes in college students using machine learning
Source: Front Public Health. 2025 Jul 9;13:1606947. doi: 10.3389/fpubh.2025.1606947 (PMC12283326; doi:10.3389/fpubh.2025.1606947)
Supplement: Supplementary file 2 [file Table_2.DOCX]

**Table 2.** Descriptive statistical results of the magnitude of positive change in depression among college students

| Variables | A positive change of one level（339,61,97%） | A positive change of two levels（168,30.71%） | A positive change of three levels （40,7.32%） | F\χ² | *p* |
| --- | --- | --- | --- | --- | --- |
|  | Mean (S.D.)\  *N* (%) | Mean(S.D.)\  *N* (%) | Mean(S.D.)\  *N* (%) |  |  |
| Sex |  |  |  |  |  |
| Male | 83(24.48%) | 36(21.42%) | 11(27.50%) | 0.911 | 0.634 |
| Female | 256(75.52%) | 132(78.58%) | 29(72.50%) |  |  |
| Age | 19.90（2.26） | 19.46（1.81） | 19.92（2.36） | 2.483 | 0.084 |
| Baseline Depression | 18.04(5.23) | 23.91(3.85) | 34.22(6.07) | 234.708 | <0.001 |
| Mother’s Care | 22.45(5.82) | 21.00(5.71) | 20.52(6.53) | 4.554 | 0.011 |
| Mother’s Autonomy | 10.01(3.69) | 9.51(4.23) | 8.92(4.56) | 1.935 | 0.145 |
| Mother’s Control | 6.63(2.86) | 6.91(3.09) | 7.62(3.05) | 2.251 | 0.106 |
| Father’s Care） | 18.92(5.59) | 17.64(5.77) | 18.00(6.00) | 3.008 | 0.050 |
| Father’s Autonomy | 10.01(3.19) | 9.76(3.30) | 8.72(4.18) | 2.806 | 0.061 |
| Father’s Control | 5.69(2.63) | 5.67(3.03) | 6.80(2.90) | 2.971 | 0.052 |
| Objective Support | 10.71(3.52) | 10.19(3.64) | 10.30(3.89) | 1.239 | 0.290 |
| Subjective support | 19.20(4.29) | 18.57(3.97) | 17.72(4.86) | 2.903 | 0.056 |
| E（Extraversion） | 49.70(11.83) | 48.75(11.83) | 47.25(12.80) | 0.960 | 0.384 |
| N（Neuroticism） | 59.71(10.78) | 62.94(9.16) | 64.25(11.57) | 7.497 | <0.001 |
| P（Psychoticism） | 51.93(10.03) | 55.02(10.90) | 58.25(11.74) | 9.703 | <0.001 |
| L（Lie scale） | 43.56(9.40) | 42.41(9.25) | 43.87(11.00) | 0.937 | 0.393 |
| Positive Coping | 20.07(5.81) | 19.03(5.76) | 17.77(6.82) | 3.785 | 0.023 |
| Negative Coping | 11.07(4.32) | 11.56(4.22) | 11.62(5.35) | 0.835 | 0.434 |
| University Personality Inventory | 26.90(9.60) | 30.73(9.10) | 34.65(12.17) | 17.114 | <0.001 |
| Somatization | 1.67(0.59) | 1.94(0.72) | 2.09(0.61) | 15.075 | <0.001 |
| Obsessive-Compulsive | 2.57(0.69) | 2.78(0.72) | 3.05(0.757) | 11.458 | <0.001 |
| Interpersonal Sensitivity | 2.33(0.76) | 2.61(0.70) | 2.76(0.88) | 11.307 | <0.001 |
| Depression | 2.23(0.73) | 2.58(0.73) | 3.00(0.81) | 26.785 | <0.001 |
| Anxiety | 2.04(0.70) | 2.32(.76974) | 2.63(0.80) | 17.026 | <0.001 |
| Hostility | 1.88(0.73) | 2.20(0.76) | 2.43(0.84) | 16.395 | <0.001 |
| Phobic Anxiety | 1.81(0.69) | 2.05(0.78) | 2.26(0.84) | 10.804 | <0.001 |
| Paranoid Ideation | 1.95(0.69) | 2.1896(0.75) | 2.31(0.80) | 8.918 | <0.001 |
| Psychoticism | 1.86(0.62) | 2.1500(0.60) | 2.39(0.82) | 20.179 | <0.001 |
| Other | 1.80(0.61) | 2.12(0.63) | 2.50(0.71) | 30.438 | <0.001 |
